# Convergent and divergent mechanisms of peroxisomal and mitochondrial division

**DOI:** 10.1083/jcb.202304076

**Published:** 2023-08-02

**Authors:** Suresh Subramani, Nandini Shukla, Jean-Claude Farre

**Affiliations:** 1 Department of Molecular Biology, School of Biological Sciences, University of California, San Diego, La Jolla, CA, USA

## Abstract

Organelle division and segregation are important in cellular homeostasis. Peroxisomes (POs) and mitochondria share a core division machinery and mechanism of membrane scission. The division of each organelle is interdependent not only on the other but also on other organelles, reflecting the dynamic communication between subcellular compartments, even as they coordinate the exchange of metabolites and signals. We highlight common and unique mechanisms involved in the fission of these organelles under the premise that much can be gleaned regarding the division of one organelle based on information available for the other.

## Teaser

Subramani et al. review the shared and unique mechanisms of peroxisomal and mitochondrial division and their dependence on subcellular organelles.

## Introduction

A distinction among eukaryotic cells is the evolution of distinct subcellular compartments, which must divide during cell division and segregate between cells (Asare et al., 2017; Klecker and Westermann, 2020; Knoblach and Rachubinski, 2015). To achieve this, the compartments such as the endoplasmic reticulum (ER), nucleus, vacuole (yeast lysosome), mitochondria, chloroplasts, and POs have evolved organelle division, segregation, and inheritance machineries for their proper distribution (Ayala and Colanzi, 2017; Barr, 2002; Boldogh et al., 1998; Fagarasanu et al., 2010; Jongsma et al., 2015; Knoblach and Rachubinski, 2015, 2016; Lowe and Barr, 2007; Miyagishima and Kabeya, 2010; Warren and Wickner, 1996; Weisman, 2006). The division of POs and mitochondria also contributes to efficient quality control processes during their autophagy-mediated removal (Mao et al., 2014; Yoo and Jung, 2018). Although POs and mitochondria have different evolutionary origins, they share a common division machinery (Imoto et al., 2020). Even more intriguing is the cross-dependence of several subcellular organelles upon each other for such division, which should not be surprising because these organelles dynamically communicate with one another, even as they exchange metabolites and signals (Cohen et al., 2018; Tamura et al., 2019).

Many excellent reviews exist on mitochondrial and/or PO dynamics, as well as the division machinery and its role in disease states (Abe et al., 2023; Carmichael and Schrader, 2022; Chan, 2020; Mahalingam et al., 2021). This review seeks to highlight both the convergent and divergent aspects of the division of these organelles and the physiological processes that they are coupled to, within the overall cellular context.

## A shared membrane fission machinery with organelle-specific receptors/adaptors

**Figure 1.**
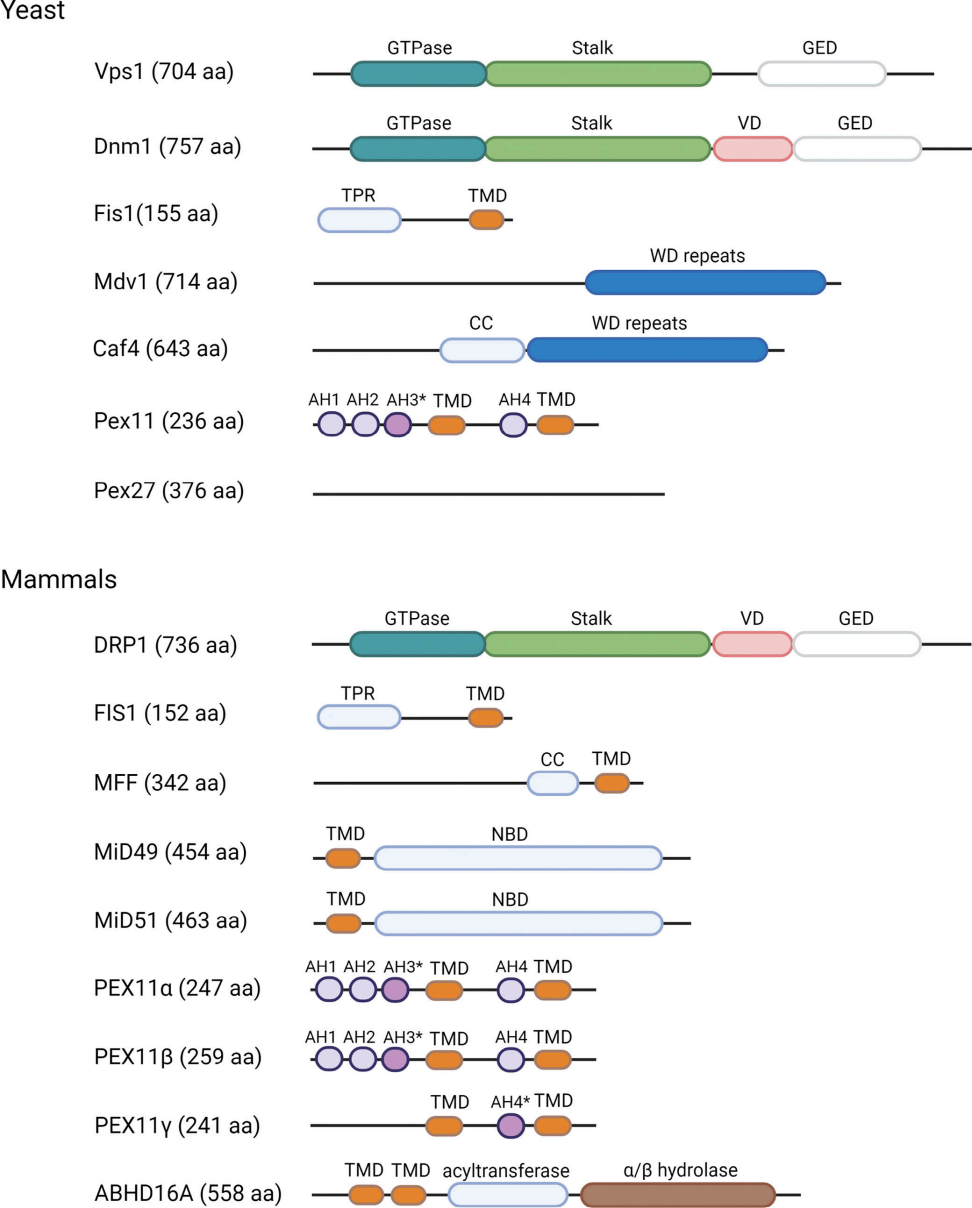
**Schematic depiction of proteins involved in the division of POs and mitochondria in yeast and mammals.** Domains with specific functions/characteristics are shown and retained in the other figures. VD, variable domain; TPR, tetratricopeptide repeat; TMD, transmembrane domain; AH1–H4, putative amphipathic helices based on the work on *P. pastoris* Pex11 (Zientara-Rytter et al., 2022); *Conserved, N-terminal AH in Pex11, termed Pex11-Amph, necessary for peroxisomal fission in vivo and for tubulation of liposomes with a lipid consistency resembling the peroxisomal membrane (Opaliński et al., 2011); CC, coiled-coil; NBD, nucleotide-binding domain. No protein domains have been reported for Pex27. Protein lengths indicate the number of amino acids (aa) in yeast (*S. cerevisiae*) or mammalian (human) proteins. DNM2 (870 aa) is not shown but it has domains similar to DRP1. All figures were prepared using Biorender.com.

POs and mitochondria share a common core membrane division machinery (Schrader, 2006) that is related to an ancient membrane division machinery found in bacteria (Osteryoung and Nunnari, 2003). It is unclear whether this commonality is reflective of a common endosymbiont origin (Gabaldón and Pittis, 2015; Osteryoung and Nunnari, 2003) or the functional relatedness of these organelles (Imoto et al., 2020). The key common proteins involved in mitochondrial and PO membrane fission are dynamin-related GTPases—dynamin 1 (Dnm1) or Vps1 in yeast, or dynamin-related protein (DRP1/DLP1) and dynamin 2 (Dyn2 or DNM2) in mammals (see Box 1 for definitions of key proteins and processes; Koch et al., 2003; Smirnova et al., 2001; Fig. 1 and Table 1).

Box 1 Abbreviations for key proteins and processes

ABHD16A, AB hydrolase domain-containing protein 16A
AMPK, AMP-activated protein kinase
Arf1, ADP-ribosylation factor 1
BNIP3, BCL2/adenovirus E1B 19 KD protein-interacting protein 3
Caf4, CCR4-associated factor 4
DNM1, yeast and mammalian dynamin-like protein
DRP1, mammalian dynamin-related protein
Dyn2/DNM2, mammalian dynamin-like protein Dynamin 2
DysF, Dysferlin domain
Fis1/FIS1, yeast and mammalian mitochondrial fission protein 1
FLNa, filamin a
FUNDC1, FUN14-domain-containing 1
INF2, inverted formin 2
Inp1, peroxisome inheritance protein 1
MARCH 5, membrane-associated RING finger (C3HC4) 5 (E3 ligase)
Mdm, mitochondrial distribution and morphology
Mdv1, yeast mitochondrial division protein 1
MFN, mitofusin
MiD49/51, mitochondrial dynamics protein of 49 and 51 KD, respectively,
Mitophagy, mitochondrial degradation by autophagy
Mmm, maintenance of mitochondrial morphology
mtDNA, mitochondrial DNA
NDPK, nucleoside diphosphate kinase
NIX, BNIP3-like
ORPL1L, oxysterol-binding protein-related protein 1L
PEX, peroxin involved in peroxisome biogenesis and/or division
Pexophagy, autophagic turnover of peroxisomes
PGAM5, phosphoglycerate mutase family member 5 (phosphatase)
PLD, phospholipase D
PMP, PXMP, peroxisomal membrane protein
PO, peroxisome
PTM, post-translational modification
RHD, reticulon homology domain
RING, really interesting gene
ROS, reactive oxygen species
TA, tail-anchored
VAP, VAMP-associated protein
VPS, vacuolar protein sorting

**Table 1.** Key proteins and their orthologs involved in peroxisomal and mitochondrial division

Protein	Ortholog	Involved in peroxisomal (P), mitochondrial (M) division, or both (P/M)	Function of protein	Reference
Organelle-specific adaptors				
ABHD16A	Unknown	M	Mitochondria-specific membrane constriction protein	Nguyen and Voeltz (2022)
PEX11-family adaptors				
ScPex11	OpPex11, KpPex11, HsPEX11α, β^a^, γ	P	Peroxisome-specific adaptor required for membrane tubulation and elongation; acts as GAP for Dnm1	Koch et al. (2010); Williams et al. (2015)
ScPex25	KpPex25, OpPex25	P	Tubulation of ER	Huber et al. (2012); Rottensteiner et al. (2003)
ScPex27	KpPex11C, OpPex11C	P	Recruitment of ScVps1	Ekal et al. (2023)
ScPex34	KpPex36	P	ScPex34 acts with the Pex11 family proteins to regulate the PO population	Tower et al. (2011)
Other adaptors				
ScCaf4		P/M in yeast	Yeast adaptor for Dnm1 recruitment	Guo et al. (2012)
ScMdv1		P/M in yeast	Yeast adaptor for Dnm1 recruitment	Motley et al. (2008)
ScFis1	MmFIS1, HsFIS1	P/M	Adaptor for Dnm1/DRP1	Koch et al. (2005); Motley et al. (2008); Mozdy et al. (2000)
HsMiD49		M	Mammalian adaptor for DNM1/DRP1 recruitment	Palmer et al. (2011)
HsMiD51		M	Mammalian adaptor for DNM1/DRP1 recruitment	Palmer et al. (2011)
HsMFF		P/M	Mammalian adaptor for DNM1/DRP1 recruitment	Gandre-Babbe and van der Bliek (2008)
HsINF2		M	ER-localized formin protein required for actin recruitment	Chhabra et al. (2009); Korobova et al. (2013)
Membrane scission GTPases				
ScVps1	KpVps1, OpVps1	P in yeast	GTPase required for organelle scission in glucose	Ekal et al. (2023); Kuravi et al. (2006)
ScDnm1	KpDnm1, MmDNM1, HsDNM1	P/M	GTPase required for organelle scission	Lee et al. (2016)
MmDNM2	HsDNM2	M in mammals	GTPase required for completion of organelle scission	Lee et al. (2016)
Accessory factors				
MmNME3	HsNME3, *C. merolae* DYNAMO1	P/M in mammals	Nucleoside diphosphate kinase	Honsho et al. (2020)

Cm, *Cyanidioschyzon merolae*; Hs, *Homo sapiens*; Kp, *Komagataella phaffii* (previously *Pichia pastoris*); Mm, *Mus musculus*; Op, *O. polymorpha* (previously *Hansenula polymorpha*); Sc, *S. cerevisiae*.

a The PEX11β isoform, working with FIS1, plays a major role in PO division in mammals (Schrader et al., 2022).

Both common and unique proteins control how Dnm1/DRP1 is recruited to these organelles (Fig. 1 and Table 1). Starting with the shared features, yeast Dnm1 is recruited to the peroxisomal and mitochondrial membranes by mitochondrial fission protein 1 (Fis1), a conserved tail-anchored (TA) protein (Mozdy et al., 2000), which is in a complex with adaptors such as mitochondrial division protein 1 (Mdv1; Tieu and Nunnari, 2000) and CCR4-associated factor 4 (Caf4; Griffin et al., 2005). Fis1 recruitment to POs depends on Pex19, a protein required for PO membrane protein assembly.

Metazoans have DRP1 and FIS1 but no homologs for Mdv1 or Caf4. Although mammalian FIS1 was thought to be the sole receptor for DRP1 during fission of POs and mitochondria, this may not be universally true (Osellame et al., 2016; Otera et al., 2010). In addition to FIS1, the TA protein mitochondrial fission factor (MFF), along with mitochondrial dynamics proteins of 49 and 51 kD (MiD49 and MiD51), which are anchored in the outer mitochondrial membrane, serve as independent adaptors that bind to and assemble DRP1 (Gandre-Babbe and van der Bliek, 2008; Otera et al., 2010; Palmer et al., 2011).

In human cells, knockout or depletion of MFF, but not FIS1, results in highly elongated mitochondria and POs (Gandre-Babbe and van der Bliek, 2008; Otera et al., 2010), suggesting that MFF is the primary adaptor protein involved in DRP1 recruitment to both mitochondrial and peroxisomal membranes. However, in MFF-deficient human fibroblasts, although POs are hyperelongated, the overexpression of PEX11β (a peroxisomal membrane protein or PMP implicated in membrane elongation and interactions with FIS1 and MFF), rescues peroxisomal, but not mitochondrial, division in a FIS1- and DRP1-dependent manner (Schrader et al., 2022). Surprisingly, the overexpression of FIS1 or DRP1 did not rescue the PO morphology in these MFF1-deficient cells. The MFF-independent recruitment of DRP1 to POs via FIS1 and limiting PEX11β might explain this result (Osellame et al., 2016). Thus, mammalian cells appear to have two independent modes of DRP1 recruitment to POs and mitochondria—one dependent on FIS1 and PEX11β and the other on MFF.

The mitochondrial division machinery is also involved in quality control of the organelle (König et al., 2021). Mitochondrially derived vesicles (MDVs) with over 100 cargo proteins have been reported in mammalian cells, and these MDV cargoes are delivered to lysosomes for turnover. These MDVs originate with the MIRO1/2-dependent formation of mitochondrial membrane protrusions pulled along microtubule filaments, followed by MiD49/MiD51/MFF-dependent recruitment of DRP1 and membrane scission (König et al., 2021).

The two modes of DRP1 recruitment for mitochondrial fission may be related to the physiological need to distinguish the biogenesis of new organelles from the degradation of parts of mitochondria for quality control. Healthy mitochondria in the mid-zone of cells divide roughly symmetrically using ER contacts, DRP1, MFF, and actin (Chakrabarti and Higgs, 2021; Kleele et al., 2021). These healthy mitochondria with normal properties such as membrane potential, ROS, and Ca^+2^ levels are segregated from smaller, dysfunctional, and abnormal mitochondrial segments located in the peripheral zone. This segregation occurs by an alternative process of asymmetric mitochondrial division involving lysosomal contact, as well as FIS1, and DRP1 (Chakrabarti and Higgs, 2021; Kleele et al., 2021). Dysfunctional mitochondrial fragments, many of which have no DNA, are delivered to adjacent lysosomes by mitophagy (Kleele et al., 2021). This important discovery may shed light on the often-confusing reports of mitochondrial division facilitating both organelle proliferation and degradation. However, a point that needs clarification is the presence of MDVs, reported by another study to be derived by MiD49/MiD51/MFF-dependent recruitment of DRP1, in the same peripheral zone where FIS1 and DRP1 act to divide and target mitochondria to lysosomes, and resolution of the conflicting information regarding whether mitophagy is involved (Kleele et al., 2021; König et al., 2021).

Like mitochondria, POs may have a process that distinguishes the division of healthy and damaged organelles. Although it is reported that PO fission is necessary for pexophagy in yeast (Mao et al., 2014), Pex27, a protein required for the recruitment to POs of Vps1, the primary GTPase required for PO division in glucose-grown yeast (Motley et al., 2008), is dispensable for pexophagy (Ekal et al., 2023). Additionally, pexophagy was only partially blocked in *fis1*Δ, *dnm1*Δ, and *vps1*Δ mutants. However, in *Ogataea polymorpha*, the removal of peroxisomal protein aggregates requires Dnm1-mediated asymmetric PO division (Manivannan et al., 2013). It would be of interest to determine whether PO division of healthy versus damaged POs is segregated by the differential involvement of Dnm1 and Vps1 in different yeast.

In addition to the common membrane fission factors, there are unique, organelle-specific receptors or adaptor proteins (Fig. 1 and Table 1). Pex11-family proteins act on POs (Koch et al., 2010), while ABHD16A is an ER-localized phospholipid lipase required for mitochondrial membrane constriction at sites where Dnm1/DRP1 subsequently assemble (Nguyen and Voeltz, 2022). During PO division, Pex11 migrates within the PO membrane to specific sites for membrane fission (Nagotu et al., 2008) and helps to activate Fis1 in the PO membrane (Joshi et al., 2012) before recruitment of Dnm1. Both peroxisomal Pex11 and ER-localized ABHD16A are constituent proteins at membrane contact sites (MCSs, see Box 2), linking the ER with either POs or mitochondria (Chu et al., 2021; Mattiazzi Ušaj et al., 2015; Nguyen and Voeltz, 2022), respectively, or connecting POs and mitochondria (Esposito et al., 2019; Mattiazzi Ušaj et al., 2015). Mitochondria also uniquely have MFF and MiD49/51, which independently recruit DRP1 (Osellame et al., 2016).

Box 2 MCSs as conduits for the exchange of lipids, ions, and/or metabolites

MCSs are sites where two membranes are present in very close proximity (about 15–60 nm), with the physical interaction resulting in the function of either or both organelles being affected (Prinz et al., 2020; Fig. 4). Components of such MCSs can include tethers that bring the two membranes in close apposition, while also creating transport channels that connect the two compartments. The organelle tethering and other (e.g., transport) functions of MCSs can be distinguished by the expression of artificial tethers linking the two organelles (Lahiri et al., 2014). The elucidation of transport functions of MCSs is complicated by their redundancy and dynamic, compensatory adjustments when one is eliminated (e.g., the absence of either the vCLAMP or ERMES causes expansion of the other MCS). In some cases, three-way MCSs have been described as involving simultaneous contacts between three organelles (Prinz et al., 2020).

POs have multiple, interorganellar MCSs (Farré et al., 2019; Wu et al., 2023) with the ER (Ackema et al., 2014; Carmichael and Schrader, 2022; Costello et al., 2017; Ferreira and Carvalho, 2021; Knoblach et al., 2013; Wu and van der Klei, 2022), mitochondria (Mattiazzi Ušaj et al., 2015; Shai et al., 2018), Golgi (Wu et al., 2023), vacuole/lysosome (Chu et al., 2015, 2021), PM (Wu et al., 2023), and LDs (Joshi et al., 2016, 2018). These contacts have the following names or acronyms and components.

PO–mitochondria or Per-Mit—Pex11 functions as a Per-Mit tether via interaction with Mdm34, a subunit of the ERMES complex, but it is unclear if this is a three-way contact between POs, ER, and mitochondria, or represents an additional PO-mitochondria contact involving ERMES subunits (Esposito et al., 2019; Mattiazzi Ušaj et al., 2015). Yeast Pex34 and Fzo1 (whose mammalian homolog is MFN1/2) are also involved in Per-Mit contacts (Shai et al., 2018).

ER–PO contacts or EPCONS—these include interactions between the major sperm protein domain of ER-localized yeast Scs2 (mammalian VAP, and VAPB) and peroxisomal Atg37 (mammalian acyl-CoA–binding domain proteins, ACBD4 and ACBD5; Costello et al., 2017; Hua et al., 2017). In mammalian cells, the interaction between VAPB and ACBD5 is negatively regulated by phosphorylation of the FFAT motif in ACBD5 at Ser269 by GSK3β (Kors et al., 2022). Interestingly, ACBD5 and VAP5 are required for the membrane expansion and elongation of POs lacking DRP1 (Hua et al., 2017), suggesting a role in lipid transport to POs prior to their membrane reorganization and division. Other contacts in yeast include one between RHD proteins, such as Pex28-32, localized at the ER and POs, and which affect PO number and size (Ferreira and Carvalho, 2021). Some of these components are necessary for Pex11 stability and, conversely, the loss of Pex11 disrupts this contact (Wu et al., 2020). Another yeast PM-PO tether, Inp1, is required for retention of POs in mother cells during their inheritance (Wu et al., 2023). Finally, mammalian POs also form membrane contacts with the ER through the interaction between peroxisomal PI(4,5)P_2_ and ER-resident extended synaptotagmin-1, 2, and 3 (E-Syts), which is involved in cholesterol transport between POs and the ER (Hua et al., 2017; Xiao et al., 2019).

LD–PO contacts are involved in de novo PO biogenesis (Ferreira and Carvalho, 2021; Joshi et al., 2016, 2018) and perhaps also in lipid exchange (Fernández-Murray and McMaster, 2016). M1 spastin is a membrane-bound ATPase localized on LDs that facilitate fatty acid trafficking to POs by two types of LD–PO contacts (Chang et al., 2019). In one mechanism, M1 forms a tethering contact with the peroxisomal ABCD1 protein. The second mechanism involves the recruitment of ESCRT-III proteins, IST1 and CHMP1B, to LDs. Additionally, upon nutritional deprivation in *Caenorhabditis elegans*, the physical contacts between LDs and POs are enhanced by the (kinesin) KIFC3-dependent movement of POs towards LDs, and the PEX5-mediated recruitment of the adipose triglyceride ligase (ATGL) to LDs for lipolysis (Kong et al., 2020).

PO–Golgi—The PMP Pex35 that regulates PO abundance is reported to physically interact with the Golgi protein, Arf1, which is a GTPase required for the formation of vesicles involved in intra-Golgi protein transport (Yofe et al., 2017).

Mitochondria–ER contacts—ERMES, MAMs, or MERCs, and EMCs—ERMES are found in yeast, but not in metazoans and plants. MAMs are mitochondria-associated (ER) membranes enriched with phospholipid biosynthetic enzymes, specifically capable of synthesizing PS, PE, and PC (Wu et al., 2016). MERCs are mammalian mitochondria–ER contacts sites, sometimes also called MAMs, containing many proteins, such as MFN2-MFN1/2 (mitofusins 1 and 2), BAP31-Fis1 (B cell–receptor-associated protein-Fis1), IP_3_R-GRP75-VDAC (inositol-1,4,5-trisphosphate receptor—glucose-regulated protein 75—voltage-dependent anion channel), and PTPIP51-VAPB or -ORP5/8 tethers (Lee and Min, 2018). In yeast, the components of the ERMES complex include two mitochondrial outer membrane proteins (Mdm10 and Mdm34), a cytosolic protein (Mdm12) and an ER protein (Mmm1), and the regulatory subunit, Gem1 (ortholog of mammalian MIRO1). Deletion of any of the three *MDM* genes listed affects the localization of Pex11 in yeast (Mattiazzi Ušaj et al., 2015).

Another ER–mitochondrial complex (EMC), composed of six ER-localized subunits (EMC1–6) plays a role in phospholipid transfer (Lahiri et al., 2014). EMC proteins interact with mitochondrial Tom5 to tether ER to mitochondria. A strain lacking multiple components of the conserved EMC shows reduced PS transfer from the ER to mitochondria, and a concomitant reduction in PE, produced mainly in mitochondria. Cells lacking both EMC and ERMES are inviable.

Finally, a third type of MCS between the ER and mitochondria uses the StART (Steroidogenic Acute Regulatory Transfer)-like domain-containing ER protein Lam6/Ltc1, interacting with mitochondrial Tom70/Tom71.

Human VPS13A and VPS13C bind to the ER, tethering it to mitochondria (VPS13A), as well as to late endosome/lysosomes (VPS13C), and lipid droplets (both VPS13A and VPS13C), and function as lipid transporters between the ER and other organelles (Kumar et al., 2018). In this context, VPS13D bridges the ER (via VAPB) to POs and mitochondria (via MIRO1 variants; Guillén-Samander et al., 2021) and its absence affects both PO biogenesis and mitochondrial morphology (Baldwin et al., 2021). It also plays a role in mitochondrial fission, downstream of DRP1, prior to mitophagy (Anding et al., 2018). Therefore, it would be worth testing if it too plays a role in lipid transfer between the ER and POs and in peroxisomal division.

Mitochondria–vacuole contacts—vCLAMP—Vacuole and mitochondria patch (vCLAMP) connects mitochondria to vacuoles (Lahiri et al., 2014). In yeast, it has vacuolar Rab GTPases, Ypt7, Vam7, the homotypic fusion and vacuole protein sorting subunit Vps39, and mitochondrial Tom40 (González Montoro et al., 2018). vCLAMP expansion caused by increased expression of Vps39 ameliorated the growth defect of the ERMES mutants on fermentable and nonfermentable carbon sources, suggesting that ERMES and vCLAMP perform some redundant functions in maintaining mitochondrial physiology (Lahiri et al., 2014). Indeed, the absence of either the vCLAMP or ERMES causes expansion of the other MCS, and elimination of both is lethal. Recently, it was discovered that mitochondria preferentially import di-unsaturated PS for subsequent conversion to PE. An analysis of the roles of the yeast ERMES, EMC, and vCLAMP in this process revealed that the ERMES and vCLAMP were more important in mediating the transfer of di-unsaturated phospholipids to mitochondria, suggesting that MCSs can be selective in the transfer of phospholipid species (Renne et al., 2022). Yeast vacuoles also have other contacts with the mitochondria (vacuolar Vps13 and mitochondrial Mcp1; González Montoro et al., 2018).

Mitochondria–LD contacts—A subpopulation of mitochondria, called peridroplet mitochondria, is attached to LDs and can be separated from cytoplasmic mitochondria (Veliova et al., 2020). The roles of these LD contacts in the organelle division are unclear at present.

Although Dnm1/DRP1 is shared in many organisms for peroxisomal and mitochondrial division, some organisms use other dynamin-like GTPases for organelle-specific division. Yeast Vps1 is involved only in PO division, being recruited to POs by its interaction with the peroxisomal proteins, Pex19 (Vizeacoumar et al., 2006), Inp1 (Fagarasanu et al., 2005), and Pex27 (Ekal et al., 2023). Like dynamin, Vps1 assembles on lipid nanotubes in vitro and interacts with membrane curvature-inducing, actin-cytoskeleton proteins, such as amphiphysin (Rvs167) and the BAR domain-containing, sorting nexin Mvp1, in vivo (Chi et al., 2014; Smaczynska-de Rooij et al., 2012). Interestingly, Vps1, but not Dnm1, plays a role in PO division in yeast cells grown in glucose (Hoepfner et al., 2001), but for cells grown in oleate, which induces POs, Dnm1 is more important, although Vps1 is still involved (Kuravi et al., 2006). Pex27, which interacts with Vps1, is specifically required for Vps1-dependent, but not for Dnm1-dependent peroxisomal fission (Ekal et al., 2023).

Why might PO division in *Saccharomyces cerevisiae* need two distinct dynamin-like proteins depending on the carbon source the cells are growing in? One possibility is that there is a redundant, back-up mechanism that is activated when one is compromised. Supporting this, Pex27, which recruits Vps1 to POs, is constitutively expressed, whereas Pex11, the PO-specific receptor/adaptor for the Fis1/Dnm1 pathway, is poorly expressed in glucose but induced in oleate. Alternatively, the two pathways might distinguish somehow the POs that need to be divided for biogenesis versus pexophagy, because, as discussed, Vps1-mediated, rather than Dnm1-Fis1-mediated, PO division plays a more important role in yeast pexophagy (Mao et al., 2014). Yet another possibility is that PO division may be coupled with the formation of other subcellular compartments, with Vps1 being involved in the fission events resulting in the creation of the endocytic compartment, as well as in Golgi-to-vacuole trafficking (Smaczynska-de Rooij et al., 2015), and Dnm1 being required for mitochondrial division (Labbé et al., 2014). These links with other compartments may involve lipid or small molecule transactions between these compartments and POs.

A similar involvement of multiple dynamin-like proteins extends to mammalian mitochondria, where two dynamins regulate division. The ubiquitously expressed, classical dynamin-2 (Dyn2 or DNM2) is a fundamental component of the mitochondrial division machinery (Lee et al., 2016). As shown in three different mammalian cell lines, Dyn2 works in concert with DRP1 to mediate sequential constriction events culminating in mitochondrial division. While DRP1 and its yeast homolog, Dnm1, assemble into spirals with a diameter of 50–60 nm, Dyn2 filaments can constrict membranes to a narrower 10–20 nm diameter to facilitate membrane fission (Kameoka et al., 2018). However, as stated in the “Membrane scission” section, this involvement of DNM2 is disputed (Kamerkar et al., 2018; Kraus and Ryan, 2017).

## A cautionary note in the search for a unified model for potentially distinct processes

The sharing and conservation of many components of the division machinery for POs and mitochondria make it tempting to think that the broad mechanistic details are universally applicable. However, while this reductionist mindset is the one that is often emphasized, it should be noted that information regarding the division of these organelles comes from different model systems that might differ based on their environment and/or metabolic state. For example, the importance of Vps1 or Dnm1, or both, for peroxisomal division depends on the growth medium, with Vps1 being critical in glucose medium and Dnm1 being more essential in oleate (Kuravi et al., 2006). Another case is that during PO turnover by pexophagy, Vps1 is more important for PO division, which is reported to be necessary for pexophagy (Mao et al., 2014), but in *O. polymorpha*, the removal of peroxisomal protein aggregates requires Dnm1-mediated peroxisomal division, followed by pexophagy of the aggregate-containing organelles (Manivannan et al., 2013). These differences in the requirement of Dnm1 versus Vps1 in yeast highlight the nuances in the division of healthy versus damaged organelles, as well as the symmetric versus asymmetric division of organelles targeted for autophagic turnover. How these two machineries are recruited to yeast POs is fundamentally different, with Pex11-family members being required for Dnm1 recruitment via Fis1 (Motley et al., 2008), whereas Vps1 is recruited to POs by Pex19 and Pex27 (Ekal et al., 2023; Vizeacoumar et al., 2006).

In an analogous manner, mitochondrial division may use distinct components to generate healthy, rather than damaged, mitochondria in different parts of the cell (Kleele et al., 2021), making it challenging and confusing to try to fit disparate processes into a unified model. Superimposed on these is the possibility that important cellular processes may necessitate in-built redundancies that predominate when one pathway is compromised. Finally, the cellular homeostasis of organelles is a balance between interconnected processes of biogenesis, turnover by selective autophagy (mitophagy or pexophagy), and organelle division (or even fusion for mitochondria), with multiple genetic, metabolic, and environmental factors determining which of these processes is more or less prevalent. In view of these variable factors, the phenotypic evaluation of cells missing a particular protein, MCS, or lipid component presumed to impact organelle division, has to be done carefully.

## Signals that determine when POs and mitochondria will divide

Growing organelles divide by fission triggered by signals intrinsic or extrinsic to the organelle. Although POs can arise by de novo biogenesis from the ER or by fission of pre-existing POs, wild-type yeast cells growing in glucose medium proliferate their POs primarily by fission, unless the cells lack POs because of a segregation defect in which case they do arise de novo (Motley and Hettema, 2007). For POs, evidence points to a signal emanating from within the POs (Guo et al., 2007). This competency for membrane fission in *Yarrowia lipolytica* is acquired only after acquisition in the PO matrix of lipid-metabolizing enzymes. When this happens, an enzyme involved in fatty acid oxidation, Acyl CoA oxidase (AOx), moves from the PO matrix to the membrane where it interacts with a peroxin, Pex16, a negative regulator of PO division that is associated peripherally with the lumenal side of the PO membrane. Pex16 binds lysophosphatidic acid (LPA), rendering this lipid unavailable for enzymatic processing. This relocation of AOx from the matrix and its association with Pex16 relieves the negative regulation of PO division by Pex16 (i.e., its restraint of LPA availability) and triggers the synthesis of phosphatidic acid (PA) and diacylglycerol (DAG) via the conversion of LPA by LPA acyltransferase to PA and further conversion of PA to DAG by PA hydrolase. The transbilayer relocation of DAG to the cytosolic face, followed by the recruitment of Vps1 and several actin cytoskeletal proteins to the PO membrane, allows fission to occur (Guo et al., 2007).

However, in mammals, the negative regulation of PO division by PEX16 is not conserved (Itoyama et al., 2012). Instead of the excessive peroxisomal proliferation observed in Pex16-knockdown in *Y. lipolytica* (Guo et al., 2007), *PEX16* knockdown has no effect on PO morphology in AOx-deficient, mammalian fibroblasts, suggesting that PO division is not regulated by PEX16–AOx complexes (Itoyama et al., 2012).

Interestingly, as described later, *PEX16* knockout in mice causes a reduction in mitochondrial abundance (Park et al., 2019). However, the mechanism by which *PEX16* affects mitochondrial abundance in mice is not likely to exist in *Y. lipolytica* because the *PEX16-KO* phenotype is rescued by plasmalogens in mice (Park et al., 2019), which are not synthesized in yeast.

In mammalian cells, PO abundance, which is primarily a consequence of division, is greatly reduced in cells with dysfunctional peroxisomal fatty acid β-oxidation (Chang et al., 1999; Funato et al., 2006; Itoyama et al., 2012), which again reflects the modulation of PO division by metabolites derived from the organelle. Docosahexaenoic acid (DHA) induces PO division by inducing the oligomerization of Pex11β (Itoyama et al., 2012), which is necessary for its function in PO division (Kobayashi et al., 2007).

Not all PO fission is driven by intraorganelle signals, because in yeast capable of growing on carbon sources other than lipids, such as methylotrophic yeasts growing in methanol, POs still divide and, in fact, the *pex11* and *fis1* mutants of *Pichia pastoris* are defective for PO fission only in oleate but not in methanol (Joshi et al., 2012). It is plausible, but untested currently, that a Pex11-independent PO division mechanism might rely more on Vps1 for division because this protein is recruited to POs, independent of Pex11, by its interaction with Pex19 and Pex27 (Ekal et al., 2023; Vizeacoumar et al., 2006). However, in another methylotrophic yeast, *O. polymorpha*, Pex11 is necessary for PO division of methanol-grown cells (Nagotu et al., 2008). Additionally, yeast or mammalian mutants incapable of matrix protein import, and therefore deficient in producing intraperoxisomal metabolites, still divide (Santos et al., 1992).

PO fission must also be responsive to other intra- and extracellular cues. The *PEX11* gene is transcriptionally upregulated in yeasts by lipids (Gurvitz et al., 2001), and both the localization and activity of Pex11 are regulated by phosphorylation (Joshi et al., 2012; Knoblach and Rachubinski, 2010). In *S. cerevisiae*, Pex11 translocates between the ER and POs in a phosphorylation-dependent manner, possibly by the action of the cyclin-dependent kinase, Pho85, whose overexpression causes the hyperphosphorylation of Pex11 (Knoblach and Rachubinski, 2010). In *O. polymorpha*, Pex11 is present over the entire PO surface but relocates during PO division and concentrates at a fission ring at the base of organelle extensions that are more easily seen in *dnm1* cells, which are blocked in the normal fission process (Cepińska et al., 2011; Nagotu et al., 2008). Alternatively, certain yeast mutants, such as *O. polymorpha pex32* cells, show reduced Pex11 expression and display a reduction in PO fission (Wu and van der Klei, 2022).

Another example of an organelle-extrinsic cue is the enhancement of peroxisomal division in the *S. cerevisiae mdm12* mutant (defective in the ER–mitochondria encounter sites, or ERMES, complex discussed later), which is not caused by an increase in Pex11 expression but is still Pex11-dependent (Esposito et al., 2019).

The role of the ERMES complex in lipid transfer between ER and mitochondria is well-established. But how might Mdm12 affect PO division through the ERMES complex? Biochemical experiments show a phospholipid-binding site located along a hydrophobic channel of the Mdm12 structure. Mdm12 might have a binding preference for glycerophospholipids harboring a positively charged head group, mainly phosphatidylcholine (PC) and phosphatidylethanolamine (PE; Jeong et al., 2016). Whether this phospholipid binding has a direct role in controlling PO fission by modulating local lipid profiles at the sites of PO division or by affecting ERMES function is unknown. The interaction between Pex11 and Mdm34 (another ERMES subunit), which is suggested to have a negative role in ERMES complex formation by repurposing the lipid transfer role of the ERMES complex from ER–mitochondria to ER–PO, might also affect PO division. Consistent with this, the knockout of Mdm10 or Mdm12 in the ERMES complex enhances PO number (Esposito et al., 2019).

The actin cytoskeleton, and signals that impact it, affect mitochondrial division. The transient, DRP1-independent assembly and DRP1-dependent disassembly of F-actin on mitochondria regulate DRP1-mediated mitochondrial fission in mammalian cells (Li et al., 2015). Mitochondria interact with the actin cytoskeleton in *S. cerevisiae*, and this interaction requires Mmm1 and Mdm10, two other subcomponents of the ERMES complex located in the ER and mitochondrial membranes, respectively (Boldogh et al., 1998). Since the yeast ERMES complex is also associated with Pex11 (Mattiazzi Ušaj et al., 2015), it is conceivable that actin might also affect peroxisomal division, in addition to actin’s known role in PO inheritance (Fagarasanu et al., 2007). Components of the ERMES complex are necessary for the correct localization of Pex11 and therefore its function (Mattiazzi Ušaj et al., 2015). In *S. cerevisiae*, a Pex11-family member, Pex25, interacts with the GTPase, Rho1, at POs and regulates the assembly of actin at the organelle (Marelli et al., 2004), and could link POs to the ERMES complex and control PO division, but this remains to be explored.

Peroxisomal division is also impacted by the cytoskeleton in mammalian cells. MIRO1-mediated membrane pulling contributes to PO membrane elongation during peroxisomal division (Covill-Cooke et al., 2020), and could be controlled by a MIRO1 variant specifically targeted to POs via interaction with PEX19 (Okumoto et al., 2018).

Signals from within mitochondria might also regulate their division, but the details are unclear. This is illustrated by the fact that mitochondria with higher respiration rates are more elongated, whereas non-respiring mitochondria become fragmented through fission (Plecitá-Hlavatá et al., 2008). Mitochondria defective in oxidative phosphorylation are also unable to proliferate POs in *P. pastoris* (Farre et al., 2022).

Mitochondrial division sites are marked by nucleoids that contain replicating mtDNA, which define the sites of ER-mediated mitochondrial division, suggesting a coupling between mtDNA synthesis and mitochondrial division during its biogenesis (Lewis et al., 2016). However, MDV formation, which occurs by mitochondrial division, does not depend on mtDNA (König et al., 2021). This signal is also not relevant for POs that have no DNA. Finally, as described in the “Membrane constriction” section, calcium signaling and lipid rearrangements may precede the marking of membrane sites for mitochondrial division.

## Cellular stress impacts the division of POs and mitochondria

Because mitochondria, and to a lesser extent, POs are involved in many aspects of energy production, it should not be surprising that different cellular stresses (due to unfolded or misfolded proteins in the cytosol or organelles, ROS or even redox imbalances) would modulate organelle homeostasis (Eisner et al., 2018; He et al., 2021). Because this is a large topic that has been reviewed elsewhere, only a few examples are cited below.

Hypoxia affects both mitochondrial and PO homeostasis. In mammalian cells, the mitochondrial protein, FUN14-domain-containing 1 (FUNDC1), recruits DRP1 to the mitochondria–ER contact sites (MERC) to allow mitochondrial fission and handling of hypoxic stress. During hypoxia, FUNDC1 on mitochondria dissociates from ER-localized calnexin at MERCs, allowing FUNDC1 to recruit DRP1 and stimulate mitochondrial fission (Wu et al., 2016). Under hypoxic conditions, USP19, an ER-resident, deubiquitinating enzyme, accumulates at the MERCs, binds to and deubiquitinates FUNDC1, which facilitates DRP1 oligomerization, GTP-binding, and GTP hydrolysis, to enhance mitochondrial division (Chai et al., 2021). This is generally followed by Parkin- and ubiquitin-dependent mitophagy. An alternative form of hypoxia-induced mitophagy is driven by ubiquitin-independent mitophagy receptors, BNIP3 (BCL2/Adenovirus E1B 19 KD Protein-Interacting Protein 3) and NIX (BNIP3-like protein), whose action is also preceded by DRP1-mediated mitochondrial fission (Sulkshane et al., 2021).

Chronic, rather than acute (Jain et al., 2020), hypoxia also enhances pexophagy in mammalian cells because when oxygen is in short supply, peroxisomal oxygen-consuming reactions, which can account for up to 20% of total cellular oxygen consumption in certain tissues, would need to be curtailed (Walter et al., 2014). In yeast, PO fission facilitates pexophagy (Mao et al., 2014), but whether PO division is necessary for hypoxia-induced pexophagy in mammalian cells is untested.

Another illustration comes from ER-stress-resistant melanoma cells, in which the induction of the unfolded protein response correlates with the downregulation of mitochondrial proteins (Baldwin et al., 2021). The concomitant induction of mitochondrial fission and mitophagy contributes to the increase in ER stress resistance. Mechanistically, the induction of the unfolded protein response results in transcriptional upregulation of an E3 ligase, membrane-associated RING finger (C3HC4) 5 (MARCH5), which facilitates ubiquitination and degradation of the mitofusin, MFN2, a protein involved in mitochondrial fusion. Reduced mitochondrial fusion tilts the homeostatic balance toward mitochondrial fission, followed by mitophagy.

## Steps in organelle fission

**Figure 2.**
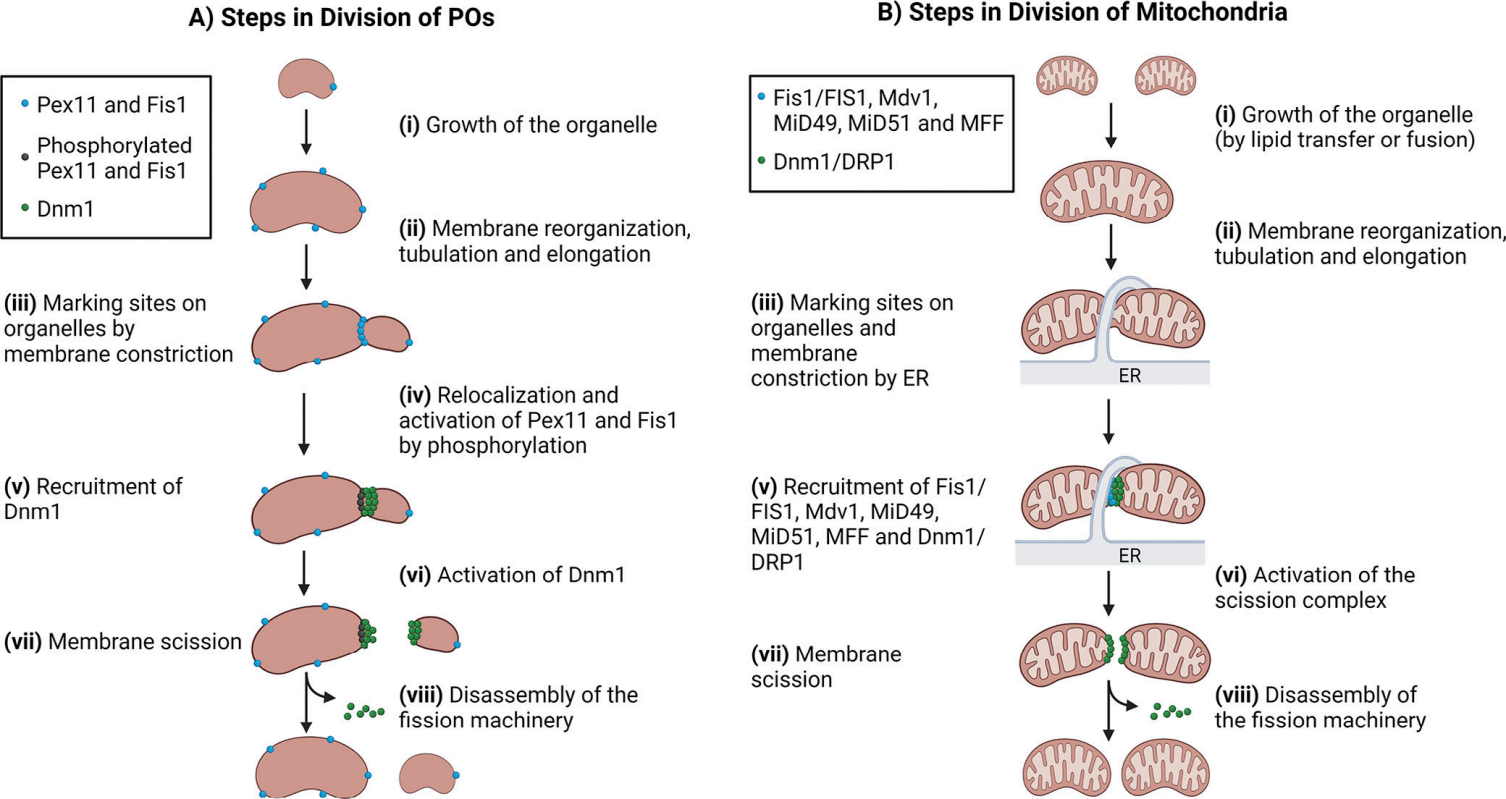
**Steps in the division of POs and mitochondria. (i)** Growth of the organelle and signaling of division. Growth of POs and mitochondria occurs by the import of membrane and matrix proteins, as well as lipids, to the organelles. Signals that activate division can be cell intrinsic or extrinsic (see “Signals that determine when POs and mitochondria will divide” section). **(ii)** Membrane reorganization, tubulation, and elongation. Membrane reorganization involves lipid rearrangements. Membrane elongation and tubulation require lipid delivery from other organelles to the site of membrane synthesis and curvature by local lipid synthesis and/or transfer using vesicular and non-vesicular processes. **(iii)** Marking sites on organelles by membrane constriction. This involves organelle-specific proteins (see text) and contact with the ER. There is evidence for both POs (Koch et al., 2004) and mitochondria (Kraus and Ryan, 2017) that membrane tubulation, elongation, and constriction occur prior to Dnm1/DRP1 assembly and action. **(iv)** Relocalization and activation of organelle-specific components, typically involving PTMs. **(v)** Recruitment and activation of Dnm1/DRP1 and formation of a double-ring contractile structure. Dynamin-like proteins involved in peroxisomal and mitochondrial division are cytosolic. **(vi)** Membrane scission. See text for details. **(vii)** Disassembly of the fission machinery (see text). **(A)** Division of POs. In yeast, the phosphorylation of Pex11 is necessary for its movement from the ER to POs (Fig. 3; Knoblach and Rachubinski, 2010), although in *P. pastoris* Pex11 is phosphorylated at the POs (Joshi et al., 2012). Pex11 phosphorylation is also necessary for its interaction with Fis1 (Joshi et al., 2012), which recruits Dnm1 to the PO membrane for division. Since Pex11 can serve as a GAP for Dnm1, the activation of Pex11 by phosphorylation (Joshi et al., 2012; Knoblach and Rachubinski, 2010), its movement in the PO membranes to the membrane constriction sites (Nagotu et al., 2008), and its interaction with Dnm1 are necessary steps in triggering peroxisomal division (Williams et al., 2015). Vps1, which is involved only in peroxisomal division in glucose-grown yeast, interacts with Pex19, Vps27, and Inp1, as well as with actin-cytoskeleton proteins like Rvs161 and Mvp1 (see text), but its role is not shown here. **(B)** Division of mitochondria. Not much is known regarding how exactly mitochondria-specific proteins, such as ABHD16A, are activated, except that calcium signaling, reorganization of the membrane lipids by ABHD16A (Fig. 3), as well as contact with the ER membrane and actin precede the membrane constriction necessary for DRP1 recruitment (Lewis et al., 2016; Nguyen and Voeltz, 2022). The role of a second dynamin (Dyn2, DNM2) in membrane scission is not shown.

Studies on POs and mitochondria have elucidated the following steps in the division of these compartments, which are discussed below (Fig. 2, A and B).

### Organelle growth and signaling of division

Organelle-intrinsic and -extrinsic signals govern when these organelles divide (see the section “Signals that determine when POs and mitochondria will divide”). While the organelle-intrinsic signals are understood partially for PO division in yeast (Guo et al., 2003, 2007) and mammals (Itoyama et al., 2012), little is known for mitochondria. Conversely, while a lot is known about organelle-extrinsic signals that modulate mitochondrial division (see “Regulation of peroxisomal and/or mitochondrial division” section), there is a paucity of studies on this topic for POs (Gurvitz et al., 2001; Knoblach and Rachubinski, 2010). Thus, research on the division of each organelle could inform that of the other.

### Membrane reorganization, tubulation, and elongation

The conserved Pex11 protein family (Fig. 1 and Table 1) is responsible for the tubulation and subsequent elongation of the PO membrane (Koch et al., 2010). However, it should be noted here that although defects in PO membrane fission components typically cause the tubulation and elongation of POs, this phenotype can also be caused by a block of lipid flow from other organelles for membrane elongation during PO division or by aberrant MCSs (Costello et al., 2017).

Pex11 proteins possess at least four amphipathic helices (AHs; AH1–AH4; Zientara-Rytter et al., 2022) with lipid-binding and membrane-bending properties (Koch and Brocard, 2011), analogous to BAR-domain proteins required for the scission of clathrin-coated vesicles during endocytosis (Suetsugu et al., 2010). In general, two types of AHs, reported in many proteins, can be involved in the induction of membrane curvature and sensing this curvature (Drin et al., 2007). Some helices favor induction of curvature because the positive charges on one face of the AH can interact with the negative polar headgroups of the membrane lipids. Yeast Pex11 and mammalian PEX11β have two such AHs (AH2 and AH3) near their N-termini (Fig. 1). In contrast, if the AH has mainly negative charges, these would obstruct its insertion into a flat, two-dimensional membrane, but could serve as curvature sensors of a previously curved membrane (e.g., AH4 two paragraphs below).

Binding of Pex11 to negatively charged liposomes via an N-terminal AH causes membrane tubulation. Additionally, the propensity of the AHs in Pex11 to oligomerize and remodel membranes is conserved from yeast to humans (Opaliński et al., 2011). As stated earlier, the oligomerization of Pex11 may be stimulated by lipids, such as DHA in mammals (Itoyama et al., 2012).

PpPex11 has a very short AH1 (6 aa long; Fig. 1). AH2 and AH3, which from several organisms have membrane tubulation properties (Opaliński et al., 2011), are involved in its dimerization (Zientara-Rytter et al., 2022). The negatively charged AH4 in Pex11 could serve as a curvature sensor to recruit the membrane fission machinery (Knoblach and Rachubinski, 2010). However, it remains to be tested whether different AHs in Pex11 confer both membrane curvature-inducing properties responsible for polarized membrane elongation (Opaliński et al., 2011; Su et al., 2018; Yoshida et al., 2015), as well as curvature-sensing properties essential for PO division.

Mitochondria synthesize cardiolipin (CL) and PE but also receive lipids from other organelles, making their membrane elongation dependent on both local lipid synthesis and interorganelle transfer. There is some evidence that mitochondria become tubulated upon incubation with DRP1 or DLP1 protein in vitro or in vivo (Yoon et al., 2001). The tubes have a diameter of ∼30 nm and are decorated with protein densities. However, as described later (“Recruitment and activation of the shared, core division machinery” section), since Dnm1/DRP1 assembles primarily at preconstricted sites on the mitochondrial membrane, it remains to be seen if the Dnm1/DRP1-stimulated tubulation represents further membrane constriction happening at a later stage of the fission process.

In the case of yeast Vps1-mediated membrane fission, Mvp1 tubulates endosomal membranes and recruits Vps1 to sites of fission (Suzuki et al., 2021). However, whether this is true for PO division in yeast has not been studied.

### Membrane constriction

Contact of the membranes with the ER and the ERMES complex defines the sites of these constrictions in mitochondria (Friedman et al., 2011; Lee and Yoon, 2014), but whether this is also the case for POs has not been studied. This preconstriction step reduces the average mitochondrial diameter from ∼300–500 to ∼150 nm to allow DRP1-oligomeric ring formation (Friedman et al., 2011). In *Aspergillus* mitochondria, calcium chelation prevents the formation of mitochondrial constrictions (Garrido-Bazán and Aguirre, 2022), suggesting that calcium signaling is necessary for mitochondrial division. An ER-localized phospholipid hydrolase, ABHD16A, which also has an acyltransferase domain, is required for mitochondrial membrane constriction (Nguyen and Voeltz, 2022). Point mutations in the critical acyltransferase motif of ABHD16A fail to rescue the formation of ER-associated mitochondrial constrictions, suggesting it alters the phospholipid composition at ER–mitochondria MCSs (Nguyen and Voeltz, 2022). ABHD16A has a hydrolase activity that converts PS to lyso-PS, which could affect local membrane lipid composition (Kamat et al., 2015). The α/β hydrolase domain of ABHD16A is necessary and sufficient for the creation of mitochondrial constrictions, whereas both the hydrolase and acyltransferase domains are required for the recruitment of fission and fusion machinery to ER-associated nodes (Nguyen and Voeltz, 2022). It is unclear whether analogous proteins govern membrane constrictions for PO division and how the ER influences this process.

**Figure 3.**
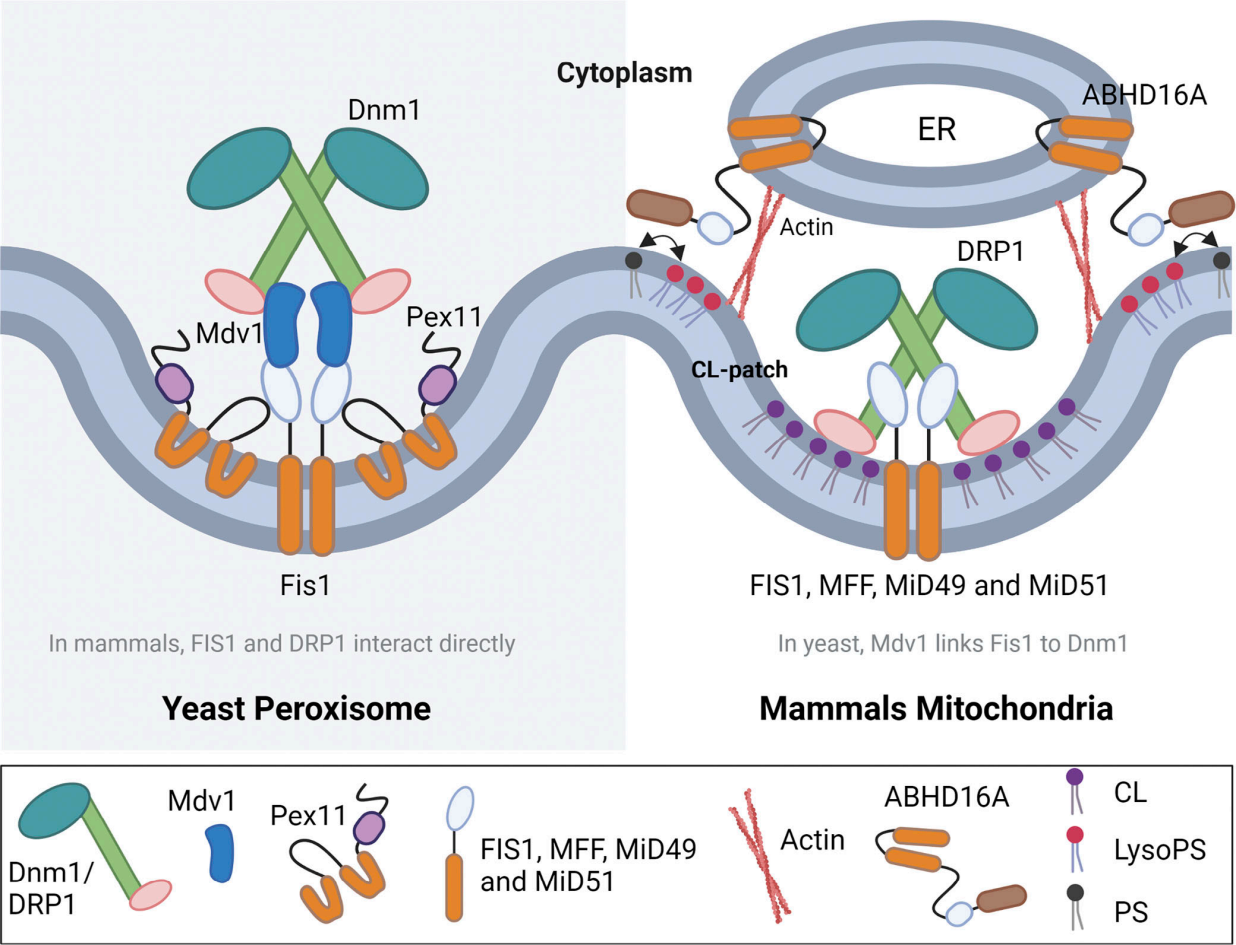
**Membrane constriction and scission steps of peroxisomal and mitochondrial division.** Color scheme for protein domains is as shown in Fig. 1. Pex11 is involved in membrane tubulation during PO division, while Fis1/FIS1, Mdv1, MFF, and MiD49/51 recruit Dnm1/DRP1 for membrane scission. In mitochondria, the hydrolase domain of ABHD16A and ER tubules helped by actin cause membrane constriction of mitochondria. The hydrolase of ABHD16A facilitates membrane lipid remodeling by the conversion of PS to lyso-PS, while the acyltransferase domain of this protein facilitates assembly of the mitochondrial fission (and fusion) machinery (Nguyen and Voeltz, 2022). Patches of the lipid CL activate DRP1 (Bustillo-Zabalbeitia et al., 2014), which is assembled at mitochondrial membrane constriction sites using FIS1, MFF, MiD49 and MiD51.

Additionally, in mammalian cells, the ER-bound inverted-formin 2 (INF2) and the mitochondrially anchored, formin-binding Spire1C proteins act together to assemble actin filaments at mitochondria–ER contact sites (Fig. 3), which mediate constriction prior to ring formation around the membrane by DRP1 (Chakrabarti et al., 2018; Korobova et al., 2013; Manor et al., 2015). The interaction of INF2 and Sprie1C likely induces the assembly of actin filaments at ER–mitochondria contact sites to drive initial mitochondrial constriction, which allows DRP1 assembly and a secondary constriction (Korobova et al., 2013). INF2 also has another role specific to mitochondria, which relates to the requirement of division for both the inner and outer membranes of mitochondria. In addition to stimulating DRP1 assembly on the mitochondrial outer membrane (Ji et al., 2015; Korobova et al., 2013), INF2 activates, through actin/myosin IIA polymerization and mitochondrial calcium influx, the fission of the inner membrane at constriction sites, independent of DRP1 (Chakrabarti et al., 2018).

Sites of mtDNA replication are associated with a subset of nucleoids and are spatially linked to ER–mitochondria contact sites in human cells (Lewis et al., 2016). These membrane contacts and nucleoids precede mitochondrial constriction and division (Lewis et al., 2016), but how these events are coordinated is unknown.

### Activation of organelle-specific division components

See factors that affect division (Table 2).

**Table 2.** Regulation of dynamin-like proteins and their receptors/adaptors involved in PO and mitochondrial division via PTMs

Organelle-specific receptor/adaptor and role	PTM	Protein implicated in PTM	Direction of regulation of division	Protein reversing PTM	References
Pex11—peroxisomal division in yeast and mammals	Phosphorylation of ScPex11-S^165^ and ScPex11-S^167^	PHO85	Positive	Unknown	Knoblach and Rachubinski (2010)
ABHD16A—mammalian mitochondrial division	Unknown				Nguyen and Voeltz (2022)
Fis1/FIS1—peroxisomal and mitochondrial division in yeast and mammals	Ubiquitination of HsFIS1	MARCH5	Positive		Yonashiro et al. (2006)
MFF—peroxisomal and mitochondrial division in mammals	Phosphorylation of MFF-S^155^_,_MFF-S^172^, MFF-S^275^	PKD1	Positive		Pangou et al. (2021)
	Phosphorylation of MFF-S^155^ and MFF-S^172^	AMPK	Positive		Toyama et al. (2016)
	Ubiquitination of MFF (multiple sites)	PARK2	Negative		Lee et al. (2019)
MiD49/Mid51—mammalian mitochondrial division	Ubiquitination of Mid49	MARCH5	Negative		Xu et al. (2016)
INF2—mammalian mitochondrial division	Farnesylation at C terminal C^1270^	Unknown	Positive		Chhabra et al. (2009)
Dynamin-related protein needed for membrane fission	PTM	Protein implicated in PTM	Direction of regulation of division	Protein reversing PTM	Reference
Dnm1/DRP1—yeast and mammalian peroxisomal and mitochondrial division	Phosphorylation of HsDRP1- S^616^	CDK1, CDK5, PKCδ, PINK1, MAPK/ERK1,2, ROCK, CaMKIIα	Positive	DUSP6, Calcineurin, PP2A	Che et al. (2022); Dickey and Strack (2011); Kashatus et al. (2015); Ma et al. (2020)
	Phosphorylation of HsDRP1- S^637^	PKA, CaMKIα, ROCK1, AMPK	Negative	PGAM5S	Cereghetti et al. (2008); Cribbs and Strack (2007); Wang et al. (2012a); Wikstrom et al. (2013)
	Phosphorylation of HsDRP1- S^693^	GSK3β	Negative		Chou et al. (2012)
	Ubiquitination	MARCH5, Parkin	Positive		Karbowski et al. (2007); Wang et al. (2011); Wang et al. (2021)
	HsDRP1 SUMOylation (8 Lys in B domain)	MAPL	Positive	SenP5	Braschi et al. (2009); Zunino et al. (2009)
		Ubp9	Positive		Figueroa-Romero et al. (2009); Harder et al. (2004)
	HsDRP1-C^644^ S-nitrosylation		Unknown		Cho et al. (2009)
	HsDRP1-T^585^, HsDRP1-T^586^ and others, O-GlcNac	OGT	Positive		Akinbiyi et al. (2021)
Vps1—yeast peroxisomal division	N-terminal acetylation	NatB	Unknown		Jiang et al. (2022)
	ScVps1-S^599^	PHO85	Uninvolved		Smaczynska-de Rooij et al. (2015)
DNM2—mammalian mitochondrial division	Phosphorylation of HsDNM2-S^764^	CDK1	Unknown		Chircop et al. (2011)
	S-nitrosylation of HsDNM2-C^86^, HsDNM2-C^607^	NOSIII	Unknown		Wang et al. (2012c)

### Recruitment and activation of the shared, core division machinery

Cytosolic Dnm1/DRP1 is recruited to premarked constriction sites on the organelle membrane, which coincide with the wrapping of the ER membrane around the organelle, where there are ER contact sites with mitochondria (Friedman et al., 2011). Mitochondrial adaptors, such as Fis1/FIS1, Mdv1, MFF, and MiD49/51 (Fig. 3), must be relocated to the constriction sites before DRP1 (Gandre-Babbe and van der Bliek, 2008; Mozdy et al., 2000; Palmer et al., 2011; Tieu and Nunnari, 2000; Yang et al., 2022).

Dnm1/DRP1 is activated by GTPase activating proteins (GAPs) and inhibited by guanine nucleotide exchange factors (GEFs). Pex11 from yeast or mammalian cells has GAP activity toward Dnm1 and DRP1 in vitro (Williams et al., 2015). Similarly, mammalian MFF bound to liposomes stimulates the GTPase activity of DRP1, whereas MiD51 inhibits it (Macdonald et al., 2016; Osellame et al., 2016). Additionally, CL is a potent stimulator of the GTPase of DRP1 (Macdonald et al., 2014; Stepanyants et al., 2015). Various posttranslational modifications (PTMs) and lipids regulate the oligomerization and GTPase activities of Dnm1/DRP1 (Table 2). The actin-binding protein FLNa binds DRP1 in mice and acts as a GEF for DRP1 in coordination with the actin cytoskeleton (Nishimura et al., 2018).

### Membrane scission

Dynamin GTPases polymerize around and constrict a variety of membranes to drive fission. The polymerization of Dnm1/DRP1 and GTP hydrolysis by its GTPase drive membrane scission (Lackner et al., 2009).

Dynamins have four distinct domains—head (GTPase or G domain), neck, stalk, and foot (variable domain; Fig. 1). The stalk domain has three separate interfaces for DRP1 dimerization and polymerization, as well as a GTPase-effector domain. The variable domain binds negatively charged lipids such as CL and PA (Low and Löwe, 2010). The DRP1 dimerization step correlates with nucleotide binding and organizes the catalytic machinery for GTP hydrolysis (Chappie et al., 2010).

The polymerization of DRP1 in the absence and presence of MiD49/51 is different. When DRP1 subunits within the MiD49/51 cofilament exchange and hydrolyze GTP, they release MiD49/51, and the DRP1 polymers shorten while curling into closed rings with an inner diameter of 16 nm (Kalia et al., 2018). This constriction might be sufficient to sever the double-membrane of mitochondria if both the outer and inner membranes are compressed together or if the inner membrane has already fused.

Mimicking the known involvement of nucleoside diphosphate kinases (NDPKs) in the action of several classical dynamins, in *Cyanidioschyzon merolae,* an NDPK called DYNAMO1 generates the GTP locally for Dnm1 function during the division of mitochondria and POs (Imoto et al., 2020). The mammalian homolog of DYNAMO1, NME3, localizes to mitochondria and POs and performs a similar function, at least during PO division (Honsho et al., 2020). In its absence, cells have elongated POs that fail to divide properly. However, the role of NDPKs in mitochondrial division has not been reported.

Mitochondrial membrane scission may require the sequential action of two dynamin-like proteins (DRP1 and Dyn2; Kuravi et al., 2006; Lee et al., 2016). In this revised view (reviewed nicely in Kraus and Ryan [2017]), the stable assembly of DRP1 oligomers and the transient association of Dyn2 with the constriction site may be required for optimal mitochondrial division. However, there are also reports that DNM2 is not required for mitochondrial or peroxisomal fission (Kamerkar et al., 2018; Kraus and Ryan, 2017).

Whether peroxisomal fission involves two dynamin-like proteins, with one playing a major role, requires further studies. In this context, although Vps1 and Dnm1 are believed to function in peroxisomal division in yeast cells grown in different media, their double deletion (*dnm1*Δ *vps1*Δ), but not the single *vps1*Δ or *dnm1*Δ deletion, completely blocks peroxisomal division (Kuravi et al., 2006). There was a greater reduction in PO number in oleate-induced *vps1*Δ cells relative to *dnm1*Δ or *fis1*Δ cells. However, a significant fraction of oleate-induced *vps1*Δ cells still contained two or more POs. Conversely, almost all cells of a *dnm1*Δ *vps1*Δ double-deletion strain contained only one enlarged PO, suggesting that both dynamins could be involved somewhat redundantly.

### Disassembly of the fission machinery

We only have partial answers for this step. Structural studies with a bacterial dynamin-like protein show that the conformational changes, necessary for oligomerization to form a ring structure around a lipid tube, are stabilized by non-hydrolyzable analogs of GTP. Nucleotide hydrolysis seems, therefore, to be coupled to polymer disassembly and dissociation from lipid rather than membrane restructuring. Based on structural similarities with other dynamins, it is plausible that disassembly of Dnm1/DRP1 is the result of GTP hydrolysis, following release of MiD49/51 as indicated earlier (Kalia et al., 2018).

When two dynamins are needed, as in the case of mitochondrial fission and possibly PO division, the DRP1 rings break up and segregate to the daughter organelles, whereas the Dyn2 ring stays with the mother organelle (Tilokani et al., 2018).

## Regulation of peroxisomal and/or mitochondrial division

### Regulation of organelle-specific Dnm1/DRP1 receptor/adaptors (Table 2)

Many components of the shared or organelle-specific division machineries are regulated transcriptionally (Cheng, 2022; Schrader et al., 2012) or by PTMs. For example, the PO-specific receptor protein Pex11 is transcriptionally regulated by Adr1 (Gurvitz et al., 2001), and by phosphorylation, likely by the cyclin-dependent kinase PHO85 (Joshi et al., 2012; Knoblach and Rachubinski, 2010). This phosphorylation is modulated in the cell cycle and reversed by an unknown phosphatase.

The localization of Pex11 is also determined by ER–PO contacts via the ERMES complex. Variants of this complex maintain ER–mitochondria and ER–PO contact sites (Mattiazzi Ušaj et al., 2015). The loss of ERMES components, such as Mdm10, Mdm12, or Mdm34, alters Pex11 localization, without affecting its expression, whereas deletion of *MMM1*, the gene encoding the fourth ER-associated component of the complex, does not result in an altered Pex11 localization or PO morphology phenotype nor does the loss of the regulatory component of the ERMES complex, Gem1 (Esposito et al., 2019). Pex11-GFP’s (and Pex25-GFP’s) localization patterns in the *mdm10*Δ and *mdm12*Δ are extremely similar but are very different from that seen in the wild-type strain. In yeast, the modulation of Pex11 localization by the ERMES complex is specific to cells grown in glucose, but not in oleate, although Pex11 is clearly required for peroxisomal division also in oleate (Mattiazzi Ušaj et al., 2015). For reasons that are not understood, the *mdm10*Δ and *mdm12*Δ mutants have more POs/cell than do wild-type yeast, and this is Pex11-dependent (Esposito et al., 2019).

PTMs are also seen for mitochondria-specific DRP1 receptors/adaptors (Table 2). Exemplifying this fact, in mammalian cells, MFF is a phosphorylation substrate for the energy sensor AMPK (Seabright et al., 2020). As a result, MFF is activated and mitochondrial fission is enhanced when energy is low in cells and this enhances mitophagy.

Another mitochondrial-specific adaptor, MiD51, has a cytosolic domain that adopts an enzymatically-dead, nucleotidyltransferase fold. This region senses ADP levels by binding dinucleotides like ADP and GDP, linking MiD51 activation to ADP levels (Losón et al., 2014; Richter et al., 2014). Surprisingly, the related adaptor, MiD49, which also has this fold, does not bind dinucleotides (Losón et al., 2015).

### Regulation of Dnm1/DRP1 activity or localization by PTMs or proteins

Several phosphorylation sites, kinases, and phosphatases have been identified as being required for DRP1 phosphorylation, and many of these events are linked to signaling pathways activated by metabolic events (Table 2).

There are two primary sites invoked in DRP1 phosphorylation, serine 616 (S616) and serine 37 (S637), in the human protein whose phosphorylation activates or inhibits DRP1, respectively. Several different protein kinases phosphorylate S616 (Ma et al., 2020; Table 2).

Dephosphorylation at this site is controlled by a dual specificity phosphatase, DUSP6, which is in the same complex as DRP1 in vivo and in vitro, and reverses the activation of DRP1 (Ma et al., 2020). In hepatocarcinogenesis models, S616 phosphorylation and activation of mitochondrial DRP1 causes its interaction with lysosomal Rab7 and the enhancement of mitochondria–lysosome MCSs to enhance mitochondrial division and mitophagy of dysfunctional mitochondria, which promotes hepatocellular carcinoma (Che et al., 2022). Conversely, dephosphorylation at this site by protein phosphatase 2A (PP2A) inhibits the p-Drp1^Ser616^/Rab7-mediated mitochondrial division and mitophagy and sensitizes cells to chemotherapeutic agents (Che et al., 2022).

In contrast, S637 phosphorylation of DRP1 inhibits its activity to promote elongation of the mitochondrial network and is also controlled by multiple kinases (Table 2). While glucose or serum starvation enhanced mitochondrial division and mitophagy, nitrogen starvation (by depletion of Gln or amino acids) caused mitochondrial elongation (Rambold et al., 2011). This nitrogen starvation inhibits mitochondrial fission both by decreasing DRP1 fission activity via reduced S616 and S637 phosphorylation and by preventing DRP1 localization to mitochondria (Rambold et al., 2011).

Phosphorylation at S637 is reversed by the phosphatases (Table 2). DRP1 is required for TNFα-induced programmed necrosis. Upon the induction of programmed necrosis in HeLa cells by TNFα, the mitochondrial phosphatase, phosphoglycerate mutase family member 5 (PGAM5), is involved in controlling mitochondrial division (Wang et al., 2012b). When necrosis is induced, PGAM5S dephosphorylates DRP1 at S637 and activates its GTPase activity due to loss of inhibition. DRP1 activation then causes mitochondrial fragmentation, an early and obligatory step for necrosis. Consistent with this, the knockdown of PGAM5S attenuates DRP1 activation.

Several other PTMs also regulate mitochondrial fission (Table 2). The membrane-bound E3 ligase MARCH5 may regulate DRP1 through ubiquitination (Karbowski et al., 2007). The E3 ligase, Mulan or MAPL, SUMOylates DRP1 and increases its association with mitochondria, thereby enhancing fission (Braschi et al., 2009), while the de-SUMOylase SENP5 reverses this (Zunino et al., 2009). Finally, NO-dependent nitrosylation of a DRP1 activates fission (Cho et al., 2009).

### Regulation of Dnm1/DRP1 by lipids

Membrane lipids also affect DRP1 fission activity. Of the various phospholipids, those facilitating bilayer formation, such as PC, PI, and phosphatidylserine (PS), are cylindrical in shape and stack easily into lipid bilayers. In contrast, the non-bilayer lipids, such as PE, CL, and PA, have conical shapes that induce membrane curvature (Osman et al., 2011). Therefore, they are likely to be involved in the membrane-bending events associated with both peroxisomal and mitochondrial division.

While the classical endocytic dynamin binds to phosphatidylinositol 4,5-bisphosphate, PI(4,5)P_2_, via a pleckstrin homology domain, DRP1 lacks a known lipid-binding domain but still binds to CL and PA, which activate and inhibit the GTPase of DRP1, respectively (Kameoka et al., 2018).

CL enhances the oligomerization of DRP1 and its oligomerization-induced GTP hydrolysis, which promotes the constriction of lipid tubules. In contrast, PA also induces DRP1 oligomerization but does not stimulate its GTPase, thereby antagonizing the effect of CL on mitochondrial fission (Tilokani et al., 2018). While CL stimulates DRP1, this interaction also reorganizes the membrane. Recombinant DRP1 induces CL clustering in synthetic liposomes in a GTP-dependent manner (Stepanyants et al., 2015). Such CL-enriched lipid domains could provide hot spots for mitochondrial division by locally activating DRP1 (Stepanyants et al., 2015). Additionally, the increased propensity of CL to transition from a lamellar, bilayer arrangement to an inverted hexagonal, non-bilayer configuration in the presence of DRP1 and GTP, creates localized membrane constrictions that facilitate fission. The CL-stimulated, GTPase activity of DRP1 is synergistically enhanced by the presence of MFF (Bustillo-Zabalbeitia et al., 2014), suggesting that CL and MFF may act together to potentiate mitochondrial division (Macdonald et al., 2016).

Since membranes of both POs and mitochondria of yeasts and plants have CL (Donaldson et al., 1972; Wriessnegger et al., 2007; Zinser et al., 1991), but surprisingly not POs in rat liver (Hardeman et al., 1990), it is plausible that CL also activates peroxisomal division. Interestingly, CL deficiency did not affect PO biogenesis and proliferation in *S. cerevisiae* (Kawałek et al., 2016). However, this result may not be conclusive because, in the absence of CL synthase, which was used to create the CL deficiency, phosphatidylglycerol (PG) accumulates in mitochondria and can partially compensate for several cellular functions of CL (Jiang et al., 2000). Mutants affecting ERMES complex subunits show reduced levels of mitochondrial PE and CL, suggesting that the ERMES structure is required for the exchange of phospholipids at ER–mitochondria contact sites (Kornmann et al., 2009).

In contrast, PA binding to DRP1 inhibits its activity. DRP1 binds to liposomes comprised of both unsaturated PA and saturated PC but not to liposomes that consist of only one or the other. This unique coincident lipid interaction of DRP1 binding to unsaturated PA and saturated PC inhibits DRP1 during mitochondrial division after its oligomerization on mitochondria (Adachi et al., 2016). Moreover, this interaction fails to activate the GTPase. PA constitutes only ∼5% of the mitochondrial membrane. It is produced in the ER and transferred to the outer mitochondrial membrane (Osman et al., 2011) through the ERMES MCS (Tamura et al., 2019). More specifically, the ERMES subunit, Mmm1, binds two phospholipids inside its hydrophobic cavity, preferentially binds glycerophospholipids, such as PC, PA, PG, and PS, and its association with Mdm12 generates a long, continuous hydrophobic tunnel that facilitates phospholipid transport (Jeong et al., 2017). PA is also generated from CL, which has mostly unsaturated acyl chains, in the outer mitochondrial membrane by a member of the phospholipase D superfamily of proteins, MitoPLD (Nelson and Frohman, 2015), and serves as both a precursor for other phospholipids (like CL) in the inner mitochondrial membrane, as well as a signaling molecule in membranes.

These findings regarding the role of DRP1 in mitochondrial division represent a gold mine that should be exploited to enhance our understanding of peroxisomal division. Despite this wealth of knowledge regarding how DRP1 is regulated to impact mitochondrial division, there is a marked dearth in our understanding of whether the same regulatory features also affect peroxisomal division. Such studies would extend our appreciation for the functional transactions that transpire between these two organelles.

### Other regulators of peroxisomal division

The conserved Pex11 protein has several related family members in *S. cerevisiae*, and this includes Pex11, Pex25, Pex27 (Rottensteiner et al., 2003; Tam et al., 2003), and Pex34 (Tower et al., 2011), whereas mammals encode Pex11α, β, and γ (Koch et al., 2010).

Yeast cells lacking either Pex11, Pex25, or Pex27 have fewer and larger POs than do wild-type cells, suggesting that they all play roles in peroxisomal division (Rottensteiner et al., 2003; Tam et al., 2003). POs are even more enlarged in cells harboring double deletions of these genes, such as *pex25*Δ *pex27*Δ, *pex11*Δ *pex27*Δ, or *pex11*Δ *pex25*Δ (Tam et al., 2003). Pex25 and Pex27 interact with each other and with themselves, but not with Pex11. Cells lacking all three genes also demonstrate a severe peroxisomal protein import defect (Rottensteiner et al., 2003). The overexpression of Pex27 induces narrow tubules where Pex27 and Vps1 assemble, resulting in dumbbell-shaped POs (Ekal et al., 2023). Such tubules are missing in *pex25*Δ cells, suggesting that Pex25 is involved in the early stages of membrane tubulation (Ekal et al., 2023).

Despite their similarity, however, the yeast Pex11-family proteins may play distinct roles. Pex25 causes PO membrane elongation during de novo PO biogenesis from the ER, whereas Pex27 acts after Pex25 by assembling on constricted PO membranes and recruiting Vps1 for peroxisomal division (Ekal et al., 2023). Consistent with this model, in glucose-grown yeast, the overexpression of either *VPS1* or *DNM1* in *pex25*Δ cells does not restore POs, whereas overexpression of *DNM1*, but not *VPS1*, does in *pex27*Δ cells (Ekal et al., 2023).

Several proteins, lipids, and MCSs regulate Pex11 activation. ScPex34, which interacts with the yeast Pex11-family proteins (Rottensteiner et al., 2003), such as Pex11, Pex25, and Pex27, but not with Vps1, is a positive regulator of peroxisomal division because its overexpression results in increased numbers of POs in wild-type and *pex34*Δ cells (Tower et al., 2011). Pex34 requires the Pex11-family proteins to promote peroxisomal division. In yeast, both Pex11 and Pex34 help to recruit the PO fission protein, Fis1, which in turn recruits Dnm1 to the membrane.

### Proteins regulating PO abundance/size by unknown mechanisms

**Figure 4.**
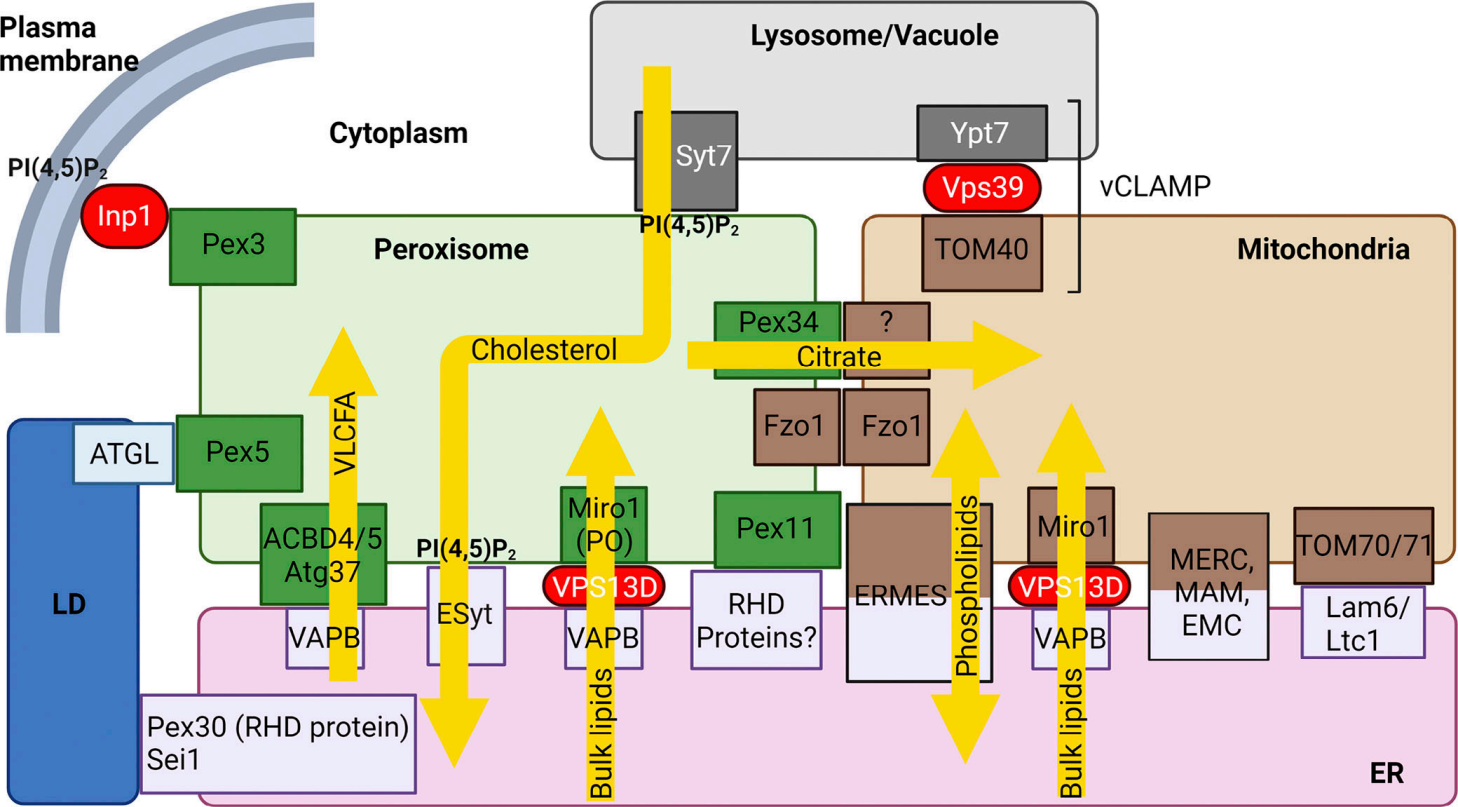
**Involvement of MCSs associated with ER, POs, mitochondria, and lysosomes in lipid and metabolite transfer between organelles (see** Box 2 **for details).** MCSs between POs and other organelles (adapted from Fujiki et al. [2022]). One example of the involvement of MCS in metabolite transport comes from the yeast ERMES complex implicated in phospholipid transfer. It serves as the mitochondrial sink for unsaturated acyl chains by mediating mitochondrial transfer of di-unsaturated PS from the ER for conversion to PE, and transports PA from the ER to mitochondria for CL biosynthesis (Renne et al., 2022). It could also be involved in metabolite (possibly other glycerophospholipids) transfer between the ER, POs, and mitochondria. Another illustration is the mammalian MCS between lysosomes, POs, and the ER, involved in cholesterol transport between the lysosomes and the ER, via the POs (Chu et al., 2021; Xiao et al., 2019). Citrate is transferred from POs to mitochondria through expanded contact sites following *PEX34* overexpression in yeast (Chalermwat et al., 2019). Very long chain fatty acids (VLCFA) are transported by the MCS involving VAPB and ACBD4/5 (Costello et al., 2017). VPS13D bridges the ER to mitochondria and POs via Miro (Guillén-Samander et al., 2021) and VPS13 transports bulk lipids, especially PC and PE (Kumar et al., 2018). PI4P is delivered to mitochondria to stimulate its division, either from lysosomes by lysosomal ORP1L, ER-localized VAP, and a three-way contact between the ER, lysosomes, and mitochondria (Boutry and Kim, 2021) or from Golgi-derived vesicles (König et al., 2021; not shown). The roles of other MCSs described in Box 2 remain to be clearly defined.

Several yeast proteins affecting PO abundance and/or size have been described, and many of these are at MCSs (Table 3). However, it is unclear whether they act directly by modulating peroxisomal division, de novo biogenesis, or even pexophagy (via metabolite transfer through MCSs; Fig. 4).

**Table 3.** Yeast proteins regulating PO abundance^a^ and size


Proteins with dysferlin domains		Deletion phenotypes (in organism listed in the first column)		
Protein	Ortholog	PO number in deletion strain	PO size/other in deletion strain	Reference
OpPex23	YlPex23	Reduced	Enhanced/most peroxisomal matrix proteins cytosolic	Wu et al. (2020)
OpPex24	YlPex24, KpPex24	Reduced	Enhanced	Wu et al. (2020)
Proteins with dysferlin and reticulon homology domains		Deletion phenotypes (in organism listed in the first column)		
ScPex28		Enhanced in oleate	Reduced and clustered in oleate	Vizeacoumar et al. (2003)
ScPex29	OpPex29, KpPex29	Enhanced in oleate	Reduced and clustered in oleate	Mast et al. (2016); Vizeacoumar et al. (2003)
ScPex30	KpPex30	Enhanced in oleate; reduced upon *PEX3* overexpression in galactose	Unchanged in oleate	David et al. (2013); Joshi et al. (2016); Mast et al. (2016); Vizeacoumar et al. (2004)
ScPex31	KpPex31	Unchanged in oleate; reduced upon *PEX3* overexpression in galactose	Enhanced in oleate	Joshi et al. (2016); Mast et al. (2016); Vizeacoumar et al. (2004)
ScPex32	OpPex32	Unchanged in oleate	Enhanced in oleate	Vizeacoumar et al. (2004)
Other proteins		Deletion phenotypes (in organism listed in the first column)		
ScPex35	None reported	Reduced in glucose and oleate	Reduced in glucose	Yofe et al. (2017)
OpPex37	HsPXMP2	Reduced in glucose, not methanol	PO segregation defect only in glucose, not methanol	Singh et al. (2020)

Hs, *H. sapiens*; Kp, *K. phaffii*; Mm, *M. musculus*; Op, *O. polymorpha*; Sc, *S. cerevisiae*.

a PO abundance could occur by one or more of the following mechanisms—increased de novo PO biogenesis or PO division, or reduced pexophagy.

*O. polymorpha* has four members of the Pex23 family (OpPex23, OpPex24, OpPex29, and OpPex32), which contain dysferlin (DysF) domains (Wu et al., 2020) that localize to the ER and affect PO abundance and size (Table 3). Pex24 and Pex32, but not Pex23 and Pex29, accumulate primarily at PO–ER contacts. The depletion of OpPex24 or OpPex32, and to a much lesser extent of OpPex23 or OpPex29, reduces the PO–ER contacts. The accumulation of OpPex32 at these contact sites is lost in cells lacking OpPex11. Since these contact sites are involved in lipid transfer between the ER and POs, clearly Pex11 has additional roles beyond the recruitment of Dnm1 and the PO fission machinery. Indeed, restoration of the ER–PO contact, using an artificial tether that juxtaposes the ER and POs, reverses the phenotypes caused by the loss of these MCSs (Wu et al., 2020).

While a *vps13Δ* mutant forms POs normally, Vps13 is essential for PO formation in a *pex11Δ* yeast mutant and is also necessary for PO formation in *pex23Δ* and *pex24Δ* cells (Yuan et al., 2022). These data have been interpreted to mean that Vps13 is crucial for PO formation in cells with reduced PO–ER contact sites and plays a redundant function in lipid transfer from the ER to POs.

*S. cerevisiae* has five proteins with DysF domains and reticulon homology domains (RHD; Table 3). These include Pex28, Pex29, Pex30, Pex31, and Pex32 (Vizeacoumar et al., 2003, 2004), which are at MCSs involving the ER, POs, and nucleus–vacuole junction (NVJ; Ferreira and Carvalho, 2021). Although these proteins primarily appear to affect the de novo biogenesis of POs, which is distinct from the division of pre-existing POs, they are considered here because they are also implicated as components of MCSs.

Yeast Pex30 targets ER–PO MCSs when bound to Pex28 and Pex32. It organizes NVJs when interacting with Pex29 and Seipin, and promotes the biogenesis of lipid droplets (LDs) independently of other family members (Ferreira and Carvalho, 2021; Wang et al., 2018). Importantly, the RHD of Pex30 is necessary for the assembly of the various Pex30 complexes. Given the role of the RHD, which is also found in other proteins like the reticulons, in membrane shaping, these results point to a mechanistic link between MCSs and regulation of membrane curvature. The integral PMPs, Pex29 and Pex30, reside in distinct regions of the ER and associate with the reticulon proteins, Rtn1 and Yop1, which contribute to ER morphology, to govern PO emergence from the ER during de novo PO biogenesis (Mast et al., 2016).

Yeast cells with single or double deletion(s) of *PEX29* and *PEX30* display an increased number of POs per cell and a decreased or unchanged average PO volume, respectively, when grown in oleic acid (David et al., 2013; Joshi et al., 2016; Mast et al., 2016; Vizeacoumar et al., 2004); however, neither deletion affects the viability of yeast cells during growth in glucose- or oleic acid–containing medium. This suggests that Pex29 and Pex30 are negative regulators of de novo PO biogenesis (David et al., 2013; Mast et al., 2016). However, another study concluded the opposite—that Pex30 and Pex31 are positive regulators of de novo PO biogenesis (Joshi et al., 2016). The reason behind this disagreement is unclear because the same yeast strains were used except for some differences in media, which might affect ScPex30 functions via its phosphorylation. Triple phosphomimetic mutants (wherein Thr60, Ser61, and Ser511 are converted to Asp) of ScPex30 result in fewer POs (Deori et al., 2022). The MAP kinase Kss1 phosphorylates Pex30 in vitro (Ptacek et al., 2005). Consistent with this, Kss1, like Pex30, is a negative regulator of PO proliferation (Saleem et al., 2008).

Pex30 associates with Scs2, a protein in the VAMP-associated protein (VAP) family, that is an important constituent of several MCSs involving multiple organelles, including POs. ER domains enriched in Seipin (Sei1) and Pex30 correspond to sites of both LDs and PO biogenesis (Joshi et al., 2018). These sites are also enriched in enzymes (like Nem1, Lro1, and Dga1) that increase the generation of DAG and triacylglycerol (TAG), which is stored in LDs as TAG-esters (Choudhary et al., 2020). Pex30 is necessary downstream of Sei1 and Nem1 to organize the ER subdomain, perhaps by using its DysF domain that is involved in lipid remodeling (Wang et al., 2018), for the recruitment of Lro1 (and presumably Dga1) to this subdomain for localized LD biogenesis (Choudhary et al., 2020). However, this idea will gain credence if the type of lipid that binds to, or is modulated by, Pex30 is discovered.

*S. cerevisiae* cells lacking ScPex35 have fewer and smaller POs (Yofe et al., 2017), but the role played by this protein is unclear. Depletion of another peroxin, OpPex37, causes a reduction in PO numbers and a defect in PO segregation between mother cells and buds only in glucose-grown cells (Singh et al., 2020). In WT cells, a single PO divides before cell division and is distributed to both mother and daughter cells, but this Dnm1-dependent division is blocked in *pex37*Δ cells, resulting in the distribution of the original mother PO to either mother or daughter, but not both. Upon introduction of human PXMP2 into *pex37*Δ cells, PO numbers, but not the PO segregation defect, are restored, indicating that this protein is a functional homolog of OpPex37.

### Role of other lipids in the division of POs and mitochondria

ER-derived phosphatidylinositols (PIs) are present in minor amounts in most intracellular membranes, including POs and mitochondria (Pemberton et al., 2020), and serve as the precursor for the synthesis of phosphatidyinositol phosphates (PIPs). Several MCSs, including contact sites between the ER and endosomes, Golgi and the plasma membrane (PM), and contact sites between lysosomes and POs, contain PIPs (Raiborg et al., 2016). At least some of these PIPs, such as PI4P, are involved in mitochondrial division (Nagashima et al., 2020).

Mitochondrial PI4P appears to be derived from both lysosomes and the Golgi through MCSs involving the Golgi apparatus and lysosomes with mitochondria (Boutry and Kim, 2021) and Golgi-derived vesicles that transport PI4P during mitochondrial division (Nagashima et al., 2020). In addition to the role of the ER in constricting the mitochondrial membrane for division, the ER also recruits lysosomes to the site of mitochondrial division through the interaction of ER-localized VAPs with the lysosomal lipid transfer protein, oxysterol-binding protein-related protein 1L (ORP1L), to induce a three-way contact between the ER, lysosomes, and mitochondria (Boutry and Kim, 2021). ORP1L is suggested to transport PI4P from lysosomes to mitochondria because the inhibition of its transfer or its depletion at the mitochondrial division site impairs fission, demonstrating a direct role for PI4P in the division process. It would be interesting to see if this delivery of PI4P from lysosomes to mitochondria is connected with mitochondrial division to generate MDVs described in the section “A shared membrane fission machinery with organelle-specific receptors/adaptors” for lysosomal delivery (König et al., 2021).

Separately, Golgi-derived vesicles also deliver PI4P to mitochondria (Nagashima et al., 2020). Microdomains containing PI4P in trans-Golgi network (TGN)–derived vesicles are recruited to mitochondria–ER contact sites to drive mitochondrial division downstream of DRP1.

In mammalian cells, PI4P can be synthesized at the TGN by a PI-4-kinase called phosphatidylinositol 4-kinase IIIβ [PI(4)KIIIβ]. Cellular depletion of the small GTPase, ADP-ribosylation factor 1 (Arf1), or its effector, PI(4)KIIIβ (Highland and Fromme, 2021), in different mammalian cell lines prevents PI4P generation and causes a hyperfused and branched mitochondrial network marked with extended mitochondrial constriction sites, reminiscent of a block in membrane fission. Depletion of Arf1, or its Arf-GEF, also causes reduced fission and aberrant mitochondrial morphology in cells from worms, mammals, and yeast (Ackema et al., 2014).

Interestingly, this link between PI4P and Arf1 could extend to POs, where the association of Arf1 with POs and its role as a negative regulator of Pex11-mediated PO fission, has been reported in oleate-grown yeast (Anthonio et al., 2009) and in mammalian cells (Anthonio et al., 2009; Passreiter et al., 1998). Yeast Pex35, which is a positive regulator of PO abundance, interacts with Arf1 and Pex11-family proteins that influence peroxisomal division (Yofe et al., 2017). However, much additional work is needed to verify this role of Arf1, PI-4-kinase, and PI4P in peroxisomal division.

#### Cross-regulation of organelle division by metabolites produced by the other organelle

An interesting example of the requirement of POs and peroxisomal metabolites for mitochondrial division comes from the study of thermogenesis in brown and beige adipocytes of mice subjected to cold conditions. The biogenesis of POs, which is responsible for fatty acid oxidation and plasmalogen (mammalian ether phospholipid) synthesis, is induced upon exposure to cold conditions. A key PO biogenesis protein, PEX16, is necessary for cold tolerance and cold-induced mitochondrial fission (Park et al., 2019). The knockdown of a plasmalogen synthesis enzyme phenocopied the effects of PEX16 inactivation on mitochondrial morphology and function. Dietary supplementation with plasmalogens increased mitochondrial copy number, improved mitochondrial function, and rescued thermogenesis in *PEX16*-AKO mice.

Human patients with genetic defects in the components of the common division machinery, such as DRP1 (also called DLP1) or nucleoside diphosphate kinase 3 (NME3, mammalian homolog of DYNAMO1; Honsho et al., 2020) affecting peroxisomal division, are also affected in mitochondrial morphology and abundance (Abe et al., 2023). NME3 localizes to both POs and mitochondria and generates GTP required for DRP1 activity. Patients with deficiencies in these factors exhibit abnormal morphology of POs and/or mitochondria.

Somewhat more surprising is the observation that patients with defects in the PO-specific division factor, Pex11β, show aberrant mitochondrial morphology and abundance. Cells from these patients exhibit defects in the metabolism of the polyunsaturated fatty acid (PUFA) DHA (C22:6; Abe et al., 2023), an essential, PO-derived lipid that induces hyperoligomerization of Pex11β, causing enhanced fission of POs in control fibroblasts (Itoyama et al., 2012). In fibroblasts from human patients deficient in DLP1, NME3, or Pex11β, DHA-containing phospholipids were decreased and, conversely, the levels of several fatty acids, such as arachidonic acid (AA, C20:4) and oleic acid (C18:1), were elevated. Whether DHA also affects mitochondrial division is less apparent.

## Summary and unanswered questions

The general theme of this review is that one can gain a deeper mechanistic understanding of the membrane fission machinery involved in the division of POs and mitochondria by paying attention to the studies involving each organelle and asking whether that applies also to the other organelle. The mechanisms of lipid reorganization, assembly and activation of the fission machinery and membrane scission are expected to be broadly similar.

In doing so, the organelle-specific and regulatory aspects will also be important to understand, especially with respect to the metabolic role of each organelle.

Because the homeostasis of each organelle is controlled by the balance between biogenesis (de novo and/or from pre-existing organelles), turnover (mitophagy or pexophagy), fission or fusion (mitochondria specific), and the impingement of overlapping and unique signaling pathways on each compartment, it is important to understand the role of organelle in the holistic context of homeostasis.

While much of the current work has been protein-centric, there is an increasing appreciation of the involvement and relevance of lipids in the membrane fission process. Because many of these lipids are made outside the POs or mitochondria, their flow via MCSs is receiving attention. However, these studies are complicated by the paucity of direct tools to follow the flow of lipids between subcellular compartments, the multiplicity and redundancy of lipid transfer pathways, and the dynamic nature of these MCSs. There remain many avenues for further research in search of answers to important questions (see Box 3). Thus, these investigations will require painstaking, quantitative analyses, often with multiple mutations blocking redundant pathways to tease out the relevant phenotypes.

Box 3 Unanswered questions

• What are the organelle-intrinsic signals that trigger peroxisomal or mitochondrial division, and how does this change with the extracellular growth environment? Is the same or different fission machinery used in response to different signals?
• What extrinsic signaling pathways impact organelle division, especially for POs, where much less is known?
• Is a single dynamin-like protein or a pair used for the completion of membrane scission in yeast and mammalian cells? Are the same proteins used during division to create healthy organelles and during disposal of damaged parts of organelles?
• What are the lipid rearrangements (in the membrane lipid domains and within the lipid bilayer) and proteins required for the creation of membrane constriction sites where the fission machinery is assembled and activated? What is the role of the actin cytoskeleton and the organelle-specific Dnm1/DRP1 receptors in membrane constriction?
• With respect to the crossregulation of the division of each organelle by other subcellular compartments, which MCSs and lipid/metabolite transport processes impact peroxisomal and mitochondrial division?
• How are the organelle-specific Dnm1/DRP1 receptors, adaptors, and fission machinery regulated by PTMs and which are common and unique to each organelle?
• How does cellular stress impact organelle division and how is organelle homeostasis maintained under these stress conditions? What is the physiological role of organelle division in the cellular response to stress?
• Recent data show, for mitochondria, a novel DRP1-independent mechanism of division (Shimura et al., 2021). How such mitochondrial division occurs is a mystery.
